# Recent trends in children's elbow dislocation with or without a concomitant fracture

**DOI:** 10.1186/s12891-019-2651-8

**Published:** 2019-06-19

**Authors:** Hanna Hyvönen, Linda Korhonen, Juuli Hannonen, Willy Serlo, Juha-Jaakko Sinikumpu

**Affiliations:** Department of Children and Adolescents Pediatric Surgery and Orthopedics Oulu University Hospital, Medical Research Centre Oulu and PEDEGO Research, Group Oulu University, PO Box 23, 90029 OYS Oulu, Finland

**Keywords:** Children, Elbow dislocation, Incidence, Fracture, Operative treatment, Non-operative treatment

## Abstract

**Background:**

The elbow is the joint that most usually dislocates in children. In contrast to the widely known recent increase in the incidence of upper-extremity fractures and their operative treatment in children, potential trends in elbow dislocation are not clear. In this study we aimed to clarify the recent epidemiology of childhood elbow dislocation, in particular the potential change in incidence and treatment.

**Methods:**

A population-based study was performed to evaluate the annual incidence and the characteristics of injury, patients and treatment. All children < 16 years of age with an elbow dislocation in 1996–2014 in the Oulu University Hospital District, Finland, were included. Elbow dislocations with and without an associated fracture were included. The mean number of children in the population at risk was 85,600, according Statistics Finland.

**Results:**

There were 104 patients with a mean age of 11.3 years (SD 2.6). The annual incidence was 6.4 (mean) per 100,000 children in 1996–2014 and no changing trend in incidence during the study period was found. Trampoline jumping was the most usual reason for the dislocations (*N* = 15, 14.4%). The majority (*N* = 73/104, 70.2%) were treated non-operatively by reduction and casting. There was no change in surgical treatment during the study time.

**Conclusion:**

In contrast to increasing incidence of upper-extremity fractures in children, there has not been a change in the incidence of elbow dislocation in children. There was no change in surgical treatment in 1996–2014.

## Background

The elbow is a complex joint, composed of three bones which all articulate, allowing it to move in three planes. Ulno-humeral joint dislocation can occur in posterior, anterior, medial or lateral directions [1, 2]. The posterior direction is the most common [3]. The trauma mechanism leading to elbow dislocation is usually similar to that of elbow fractures: a fall on an outstretched hand [4]. Dislocation is often accompanied by bone fracture, while avulsion of the medial epicondyle is the most common concomitant injury [1]. Other bone fractures associated with the dislocation are at lateral humeral condyle, radial head, olecranon and coronoid process of the ulna [5]. Prompt reduction and external splint immobilization of a simple elbow dislocation are associated with good results [2, 5–8]. A displaced fracture, however, may indicate surgical fixation to hold the joint congruent [3, 5, 9].

Of all joints, the elbow is the most common in children to become dislocated [5]. The annual rate of elbow dislocation is 3–6% of all elbow injuries in children [10]. Nevertheless, epidemiological studies concerning childhood elbow dislocation are extremely sparse: to our knowledge the only research on the annual incidence in an unselected population is by Josefsson and Nilsson, who reported 178 dislocations in adults and children in 1971–1982 in Malmö, Sweden [11]. The childhood fracture pattern has changed during the last 40 years. There is no understanding of the recent incidence and potential change in incidence of elbow dislocation in children. In contrast, many other paediatric injuries in the upper extremities have been found to have increased in recent years [12, 13]. For example, forearm shaft fractures have increased fourfold in the last decade and supracondylar humeral fractures by 30% at the same time [12, 14, 15].

This study was performed to analyse the incidence and potential changing trends in elbow dislocation in children. Associative fractures, particularly in the medial epicondyle, were to be analysed. We hypothesized that elbow dislocation in children has increased in recent years, similarly to many upper-extremity fractures.

## Methods

### Study design

A population-based study was performed and all acute traumatic elbow dislocations diagnosed in children < 16 years of age in the geographic district of Oulu University Hospital, Finland, between 1996 and 2014 were included. This is the only hospital in this region treating paediatric fractures and dislocations. Patients treated primarily at primary health-care level were also included because their follow-up visits occurred at the study institution. Hospital records were reviewed by the first author (HH), who was not involved in the original treatment of the patients. All patients had standard radiographs (anteroposterior (AP) and lateral views) obtained on the day of hospital admission. The radiographs were re-evaluated to confirm the diagnosis. There was a total of 104 patients. The injury mechanism, the side of the injury, gender, direction of the dislocation in radiographs, treatment method and operative details were noted. The initial displacement of the potential fracture was determined as the greatest perpendicular distance of the fragments in AP or lateral views.

### Primary and secondary outcomes

The incidence and the potential change in annual incidence was the primarily outcome variable, assessed by using the annual population at risk in the study area according to Statistics Finland. In our study the child population varied from 83,800 to 88,100 during the study time. In order to determine the potential change in the annual incidence between the beginning and the end of the study period, the annual incidence was determined for every single study year. The purpose was to find out if there is any increasing or decreasing trend in elbow dislocation. Possible re-dislocation during the follow-up time was taken as a complication rather than a new dislocation.

As to the secondary outcomes, the characteristics of the patients and injuries were evaluated in details. The results were based on the situation at the last follow-up visit at the paediatric trauma unit and determined by the treating surgeon. Flynn’s criteria provides a summary of both short-term functional (elbow range of motion, ROM) and cosmetic (carrying angle) results. Loss of movement and loss of carrying angle were evaluated separately by normal manner, first by visual evaluation and thereafter, if found to be abnormal, by using a goniometer. Change of motion range and/or carrying angle were classified in four groups: excellent referred to 0–5° of loss of carrying angle or ROM, good 5–10°, fair 10–15° and poor > 15° degrees [16]. Satisfactory comprised excellent and good, while unsatisfactory comprised moderate or poor. Any complications other than slight temporary sequelae at the last follow-up visit (erythema, dryness or unevenness of the skin, tingling) were taken as unsatisfactory overall outcomes.

### Statistical analysis

The annual incidence was reported in terms of 100,000 children at risk. Year by year differences in incidence were analysed by using StatsDirect software (SND test for independent variables). The injury mechanism was known for all 104 patients as well as the side of the body, gender, direction of the dislocation, treatment method and operative details regarding the number of K-wires used in fixation. The Chi-square test is a statistical test used in analyzing the categorical variables. In this study, the Chi-square test was used to analyze if there was a difference in a categorical variable concerning the outcome of the injury (satisfactory vs. unsatisfactory) between operatively vs. non-operatively treated patients among the cases who suffered from medial epicondyle fracture. The analyses for short-term outcome were performed by using Chi-square test and Fischer’s exact test for small groups (< 5 cases). The threshold of statistical difference was set at *P* < 0.05 (5%). The data were analysed by using StatsDirect Statistical Software, version 3.1.20 and IBM SPSS software, version 23.

## Results

### Incidence of elbow dislocation

The mean annual incidence of elbow dislocation was 6.4 per 100,000 children in 1996–2014. There was no trend in incidence during the study period. The incidence rate of 10.3 in the first 2 years (1996–1997) did not differ significantly from that of 9.1 in the last 2 years (2013–2014) (*P* = 0.791) (Table 1).

**Table 1:** The annual population at risk, number of elbow dislocations in children < 16-years of age and the annual incidence of the elbow dislocations per 100.000 children at risk

Year	Population at risk	Number of elbow dislocations	Annual Incidence
1996	87,300	3	3.44
1997	86,600	6	6.93
1998	85,600	4	4.67
1999	84,900	3	3.54
2000	84,500	8	9.47
2001	84,200	9	10.69
2002	84,100	6	7.14
2003	83,800	6	7.15
2004	84,300	9	10.68
2005	84,500	9	10.64
2006	85,000	8	9.41
2007	85,200	5	5.87
2008	85,300	4	4.69
2009	85,700	6	7.00
2010	86,200	2	2.32
2011	86,500	6	6.93
2012	87,300	2	2.29
2013	87,700	2	2.28
2014	88,100	6	6.81

### Patients and injury mechanism

The mean age of the patients was 11.3 years (range 3.5–15.9, standard deviation (SD) 2.6). There were 70 males (67.3%) and 34 females (32.7%). All dislocations were unilateral. Half (*N* = 54, 51.9%) of the patients had the injury on the left side. Trampoline jumping was the most frequent cause of elbow dislocation, but down-hill skiing, snowboarding and playground activities were other common causes (Table 2).

**Table 2:** Causes for pediatric elbow dislocation

Trampolining	14.4%	(15)
Down-hill skiing, snowboarding	13.5%	(14)
Playground, swing, jungle gym etc.	13.5%	(14)
Traffic injuries	10.6%	(11)
Athletics	8.7%	(9)
Rollerskating, skateboarding	8.7%	(9)
Horseback riding	6.7%	(7)
Gymnastics	4.8%	(5)
Falling on ice	2.9%	(3)
Undefined falling	16.3%	(17)

### Injury type and treatment

Posterior dislocation was the most common type of injury (*N* = 65, 62.5%). Other dislocation types were posterolateral (*N* = 23, 22.1%), posteromedial (*N* = 11, 10.6%), lateral (*N* = 2, 1.9%), medial (*N* = 1, 1.0%) and anterior (*N* = 2, 1.9%). No open dislocations were found. A slight majority (*N* = 58, 55.8%) of the patients had pure elbow dislocation and 46 (44.2%) patients suffered from an associated fracture. It was most commonly the medial epicondyle that was fractured (*N* = 30/46, 65.2%). The lateral epicondyle was fractured in five, proximal head of the radius in six patients, olecranon in two, forearm shaft in one and coronoid process of the ulna in one patient. One patient had a fracture in both the medial epicondyle and radius head and another one had both olecranon and radius head fractures. The initial displacement of the fragment ranged from 0 mm to 35 mm (mean 13.3 mm, SD 11.6 mm). There was no ulnar nerve incarceration or injury in this study population. The median follow-up time was 4 weeks and the mean follow-up time was 15 weeks (range 1 to 109 weeks).

There were two types of treatment after successful reduction of the elbow dislocation: non-operative or operative. All but one of the patients were treated by closed reduction (*N* = 103/104, 99.0%) and one was treated by open reduction. Most patients were treated on the day of the injury (*N* = 97, 93.3%) but primary treatment was delayed in six cases (5.8%) because closed reduction did not succee after the out-hospital visit. K-wires were used as an operative treatment method for all patients who underwent surgery (*N* = 31). There were 46 patients with an elbow dislocation and an associated fracture: 31 of them were operated and 15 were non-operatively treated. Thus, the majority of the all patients (*N* = 73/104, 70.2%) were treated non-operatively. Most dislocations (*N* = 95, 91.3%) were immobilized by means of an above-the-elbow cast, but nine (8.7%) were treated by means of collar and cuff immobilization after reduction.

Paracetamol and non-steroidal anti-inflammatory drugs (naproxen) were usually used analgetics in the study institution, in connection with elbow dislocation.

### Outcomes of associated medial epicondyle fractures

**Fig. 1:**
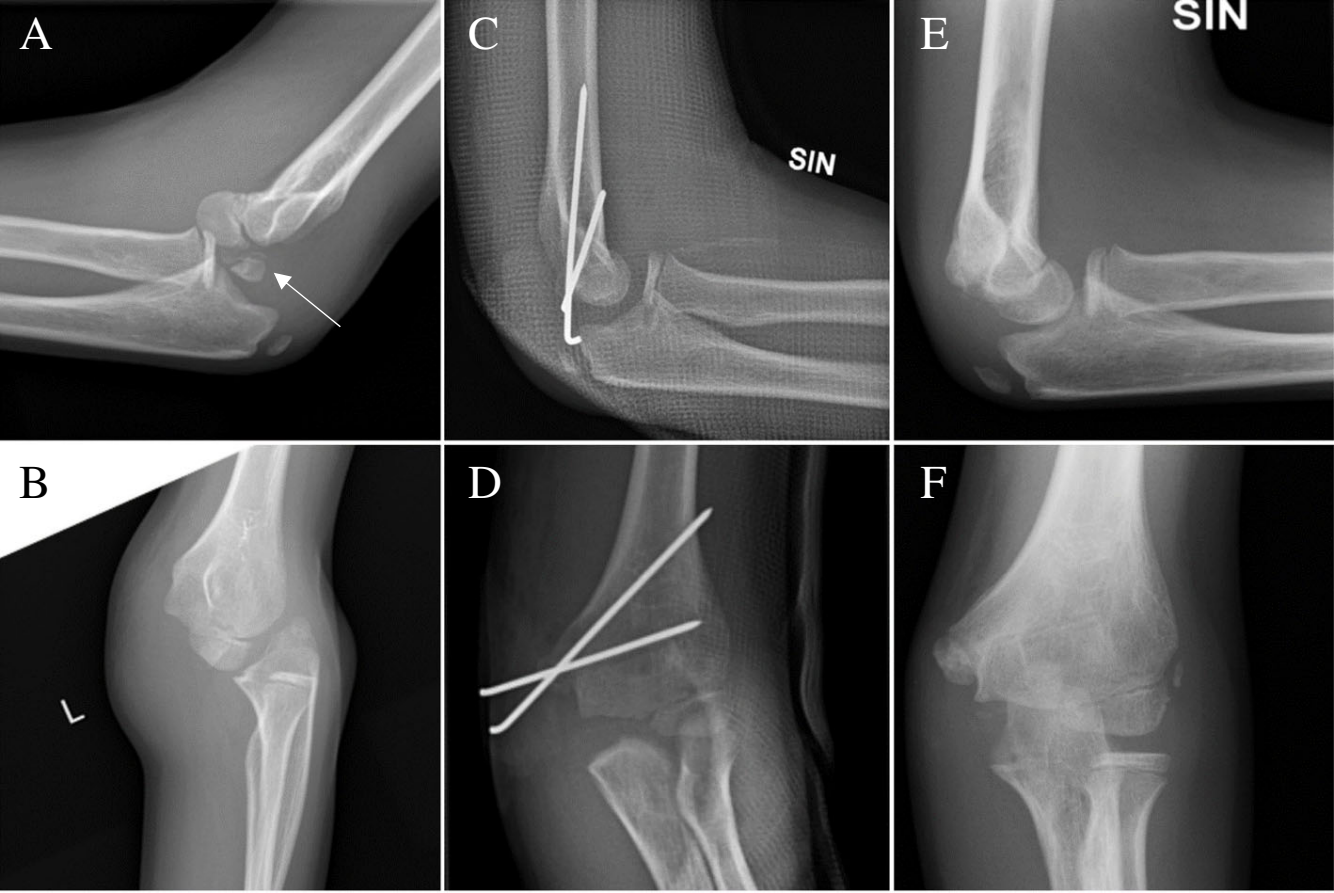
A Boy, 10.8 years old, was injured during trampoline jumping. Lateral (**a**) and anterior-posterior/oblique (**b**) radiographs of the left elbow showed a posterior-lateral dislocation and an avulsion of medial epicondyle (the arrow). Closed reduction was carried out in an emergency unit immediately and the fracture was fixed with K-wires (**c**−**d**). The elbow was immobilized with an above-the-elbow cast for 31 days. In the last follow-up visit the medial epicondyle was in good reduction. Functional and cosmetic results of the left elbow were satisfactory (**e**−**f**)

A medial epicondyle fracture (*N* = 30/46, 65.2%) was the most common associated fracture, most usually requiring surgical treatment: 22 of them (73.3%) were operatively fixed. A majority (81.8%, *N* = 18/22) of the operatively treated patients had a satisfactory outcome while 62.5% (*N* = 5/8) of the non-operatively treated patients had a satisfactory outcome (*P* = 0.345) (Fig. 1).

There was no difference in outcomes between the injury types, even in case where trampoline jumping was compared to other recreational background factors (*P* = 0.104). The association between the injury type and outcomes was separately studied among the patients with or without a concomitant fracture, nevertheless, no statistically significant difference in outcomes was found between these groups.

## Discussion

The mean incidence of elbow dislocation was 6.4 per 100,000 children during the long study period (1996–2014). There was no change in the incidence. The findings are important because the previous understanding of the epidemiology of childhood elbow dislocation is markedly limited and based on the epidemiological findings four decades ago. However, our findings are surprising, keeping in mind that upper-extremity fractures rather than lower-extremity fractures have increased in children recently. For example, forearm fractures (distal, middle and proximal) increased by 31% and upper-arm fractures by 39% over 22-year period (1983–2005) in Helsinki, Finland [13]. The injury mechanism of elbow dislocation is mostly the same as that of forearm or elbow fractures: a fall on the arm. Therefore, it remains unclear why fractures in the elbow region have increased steadily but the same trend is not seen with elbow dislocations. There may be not only environmental reasons, for example changed recreational activities, but also biological reasons behind this finding: biological changes may have affected bone structure rather than ligaments and may have resulted in an increasing incidence of fractures in children. Childhood bone fractures tend to be multifactorial in nature and both external and internal factors may exist. Bone characteristics like bone mineral content and bone size are lower in children and adolescents who suffer from fractures. It has been found out that low bone mineral density predicts new fractures [17, 18]. Maternal smoking is associated with increased risk of childhood fractures [19]. Similarly, we suppose that there are both external and internal causes resulting in joint dislocations, too, in children patients.

An associated fracture was found in 44% of the patients, which is less than previously reported: Carlioz and Abols reported associative fractures in 64% [20] and Rasool in 75% [1] of patients. Di Gennaro et al. reported a bone fracture in connection with dislocation in 67.5% of cases [7]. However, we utilized a population-based study setting and radiographs were available for all; our results are certain. A medial epicondyle fracture was the most usual associative fracture, which is in accordance with reports in the literature [1, 7]. We therefore analysed medial epicondyle fractures (*N* = 30) separately, revealing satisfactory outcomes in 82% of surgically treated patients. In turn, 63% of non-operatively treated patients had satisfactory short-term outcomes. The difference did not reach statistical significance and the groups were small. However, this trend is in line with reports in current literature [21]. Operative fixation has been found to be superior to non-operative treatment in cases of medial epicondyle fracture among patients with elbow dislocation according to a recent study of 498 patients [22]. Regardless of fracture union rate, clinical or functional disabilities seem to be minimal after non-operative treatment of medial epicondyle fracture with or without associated elbow dislocation [8]. In general, the aim of operative treatment in cases of elbow dislocation with an associated fracture is to maximize an early return to daily mobilization and high-level activity [22].

There are some limitations in this study. Only short-term outcomes of medial epicondyle fracture could be determined, although, long-term outcomes would be important as regards childhood trauma, justifying further research with longer follow-up periods [23, 24]. Despite the population-based study setting, there may have been a few isolated cases that were treated elsewhere, for example during a holiday trip, and were therefore not enrolled. As another weakness, the fractures were diagnosed by radiographs, possibly resulting in the fact that not all osteochondral fractures were found. Magnetic resonance imaging was not performed in the cohort [25] due to practical reasons [8]. As a strength, this is the only study concerning the incidence and recent trend in childhood elbow dislocation in a normal child population since the 1980s, to best of our knowledge. The patients were children and adolescents of < 16 years of age in a particular geographical study area comprising both rural and urban regions. An appropriate population-at-risk size was determined by using official statistics and the incidence numbers are precise. The follow-up time was not long but sufficient to demonstrate the immediate results and complications, in particular the need of reoperation. The original radiographs of all patients were re-reviewed for the purpose of the study.

## Conclusion

The mean incidence of elbow dislocation was 6.0 per 100,000 in children and showed no changes in trend in 1996–2014, in contrast to paediatric fractures in that area. Trampoline jumping was the most usual reason for elbow dislocation, which was not benign injury in nature in all cases.

## Data Availability

The dataset analysed during the current study are not publicly available due individual privacy but are available from the corresponding author on reasonable request.

## Abbrevations

ROM: Range of motion
